# HIV and the risk of tuberculosis due to recent transmission over 12 years in Karonga District, Malawi^☆^

**DOI:** 10.1016/j.trstmh.2009.03.013

**Published:** 2009-12

**Authors:** Rein M.G.J. Houben, Amelia C. Crampin, Kim Mallard, J. Nimrod Mwaungulu, Malcolm D. Yates, Frank D. Mwaungulu, Bagrey M.M. Ngwira, Neil French, Paul E.M. Fine, Judith R. Glynn

**Affiliations:** a Karonga Prevention Study, Malawi; b Infectious Disease Epidemiology Unit, London School of Hygiene and Tropical Medicine, Keppel Street, London WC1E 7HT, UK; c Pathogen Molecular Biology Unit, London School of Hygiene and Tropical Medicine, London, UK; d Mycobacterium Reference Unit, Health Protection Agency, London, UK

**Keywords:** HIV, Tuberculosis, *Mycobacterium tuberculosis*, Molecular epidemiology, Transmission, Malawi

## Abstract

Tuberculosis (TB) patients with strains common to other recent cases (‘clustering’) suggest recent transmission. HIV status and age may affect proportions clustered. We investigated TB clustering by HIV and age in a population-based study in Malawi. Among 746 patients, HIV infection increased the proportion clustered. Sex-period-adjusted odds ratios for the association of HIV and clustering were 1.26 (95% CI 0.4–4.1) for ages 15–25 years, 1.40 (0.9–2.3) for 25–50 years and 10.44 (2.3–47.9) for >50 years and remained stable over two periods examined. These results suggest that HIV increases the proportion of TB due to recent transmission in the elderly.

## 1 Introduction

Tuberculosis (TB) is the main cause of death in HIV-positive patients from low-resource settings, and HIV infection increases an infected person's risk of active TB disease from 10% in their lifetime to 10% annually.^1^ However, the relative effect of HIV on TB due to recent or past infection is not known.^2^

Active TB disease follows recent infection or reinfection with *Mycobacterium tuberculosis* (Mtb), or reactivation of a latent Mtb infection.^3^ It is assumed that cases with identical strains are likely to represent recent infection and that each ‘cluster’ of cases with common strains contains one index case due to reactivation.^4^ The number of cases in clusters, minus the number of clusters, as a proportion of all cases, thus gives an estimate of the proportion of TB due to recent transmission (the ‘n − 1’ method).^4^

We have previously shown that HIV increased the proportion of TB cases clustered in rural Malawi, but only among older adults.^5^ Here we repeat the analysis, including four more years of data to investigate the robustness of the finding and the stability of this association between HIV and TB clustering over time.

## 2 Materials and methods

The Karonga Prevention Study in rural Malawi has been collecting TB molecular epidemiological data since late 1995, with results now available up to October 2007. The methods have been described previously: all patients with TB in Karonga District (population ∼250 000) were included and were asked to undergo HIV testing.^6^ Isolates from all culture-confirmed TB cases were typed by *IS*6110 RFLP fingerprinting.^5^

After excluding possible cross-contamination, TB cases were considered clustered if another patient had an identical Mtb strain in the previous 4 years, based on a previous study in Karonga showing that maximum clustering was reached within 4 years.^5^ Cases in the first 4 years were used to determine cluster status of subsequent cases, but were then excluded from the analysis. This retrospective clustering^5^ with a fixed time window gives an estimate of ‘n − 1’ clustering, and allows comparison between time periods that is unbiased by the total duration of the study.

For the main statistical analysis two periods were compared: using data that have previously been reported (October 1999–March 2003)^5^ and new data (April 2003–October 2007). Cases were stratified into three age groups (15–25, 26–50 and >50 years).

Multivariate logistic regression (Stata v.10; Stata Corp., College Station, TX, USA) was used to calculate age and period-stratified odds ratios (ORs) for the association of HIV with TB clustering, adjusted for sex. Interactions were assessed through likelihood ratio tests.

To test the robustness of the results we repeated the statistical analyses, including cases from the first 4 years, comparing either two 6-year periods or three 4-year periods. We also explored the impact of restricting clustering to time windows of either 1 or 2 years,^5^ or expanding clustering to any case with an identical Mtb strain in the study period, both retrospectively and prospectively.

## 3 Results

DNA fingerprints were available for 1630/1968 (83%) of all culture-positive cases between late 1995 and October 2007. The median age was 35 years (range 17–85 years) and 767/1630 (47.1%) were male. Excluding the cases in the first 4 years, 705/1031 (68.4%) of cases were clustered with a case in the previous 4 years, and 493/746 (66.1%) were HIV-positive.

**Figure 1:**
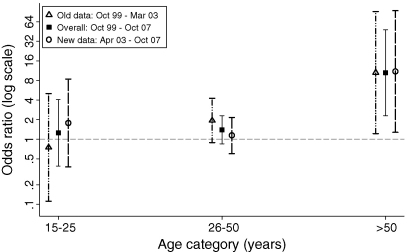
Odds ratios for tuberculosis clustering according to HIV status, by study period. All odds ratios are stratified for age and adjusted for sex. Overall odds ratios are adjusted for study period. Error bars show 95% CIs for the odds ratio.

An interaction was shown between age and HIV in the overall model (*P*-value LR test = 0.05) and both study periods (*P*-value LR tests = 0.1). The models (Figure 1) show that in both time periods and overall the ORs in the young and middle age categories were not statistically different from 1. However, in the older age group HIV infection was associated with increased clustering. This pattern was the same in all sensitivity analyses.

Overall, in the two periods together, the proportion retrospectively clustered among the HIV-negative was 36/49 (73%), 77/122 (63%) and 41/82 (50%) in age groups 15–25, 26–50 and >50 years, respectively. The equivalent figures for the HIV-positive were 36/45 (80%), 297/411 (72%) and 31/37 (84%).

## 4 Discussion

This paper shows that the association we reported previously between HIV and TB clustering, at least in the elderly, persists and is thus very unlikely to be due to chance. Combined with the observation that HIV-positive TB cases are on average less likely to be the source of Mtb transmission,^1^, ^7^, these results strengthen the hypothesis that HIV mainly increases the risk of TB disease due to recent (re)infection. Further work is needed to study how antiretroviral therapy (ART) affects the association between HIV and TB clustering. ART has been available in Karonga since June 2005 and has already been shown to reduce mortality in the population.^8^

Although efforts to control TB in settings with generalized HIV epidemics should always be multifaceted, our results suggest that measures aimed at reducing TB disease attributable to recent transmission could be more effective in reducing TB incidence than concentrating on those with latent infection.

## Funding

The study was funded by The Wellcome Trust UK and the British Leprosy Relief Association.

## Conflicts of interest

None declared.

## Ethical approval

The study was approved by the National Health Sciences Research Committee of Malawi (reference numbers HSRC-64-96, NHSRC-01-38, NHSRC 424) and the Ethics Committee of the London School of Hygiene and Tropical Medicine, UK (reference numbers 384, 745A, 5067).

## Authors’ contributions

JRG, RMGJH and ACC contributed to the conception and design of the study; RMGJH, ACC, KM, JNM, MDY, FDM, BMMN, NF, PEMF and JRG all acquired data or actively participated in data-management activities; RMGJH performed the statistical analysis and drafted the report. All authors commented on the manuscript, suggested revisions and read and approved the final version. RMGJH and JRG are guarantors of the paper.
